# Phenocopies of 22q11.2DS: revealing genetic diversity in clinically suspected 22q11.2 deletion syndrome

**DOI:** 10.1186/s40348-026-00236-1

**Published:** 2026-04-30

**Authors:** Fanni Szumutku, Anna Lengyel, Éva Pinti, Ilona Kun, Krisztina Németh, Tünde Abonyi, Fanni Gál, Seung Woo Ryu, Yongjun Song, Anikó Ujfalusi, Krisztina Kádár, Eszter Jávorszky, Kálmán Tory, Éva Kis, Vera Goda, Gergely Kriván, Árpád Ferenc Kovács, Irén Haltrich

**Affiliations:** 1 Tűzoltó Street Department, Pediatric Center, Semmelweis University, Budapest, Hungary; 2 3billion Inc, Seoul, South Korea; 3 Department of Medical Genetics, Faculty of Medicine, University of Debrecen, Debrecen, Hungary; 4 Heart and Vascular Center, Semmelweis University, Budapest, Hungary; 5 MTA Center of Excellence, Pediatric Center, Semmelweis University, Budapest, Hungary; 6 Gottsegen National Cardiovascular Center, Budapest, Hungary; 7 Central Hospital of Southern Pest National Institute of Hematology and Infectious Diseases, Budapest, Hungary; 8 Department of Pathology and Experimental Cancer Research, Semmelweis University, Budapest, Hungary

**Keywords:** Exome sequencing, 22q11.2 deletion syndrome, Phenocopies of 22q11.2 deletion syndrome, CNV analysis, DiGeorge syndrome

## Abstract

**Background:**

Although 22q11.2 deletion syndrome (22q11.2DS) is one of the most common microdeletion syndromes, a substantial proportion of patients with clinically suspected 22q11.2DS (clin22q11.2) remain without a definitive diagnosis. While CNVs other than the typical 22q11.2 deletion have been identified in patients with clin22q11.2 (defined here as phenocopies of 22q11.2DS, or phen22q11.2), SNVs associated with phen22q11.2 are less well defined.

**Results:**

We aimed to investigate genetic variants associated with phen22q11.2 to achieve definitive diagnoses and improve clinical management in the clin22q11.2 cohort, while also comparing the phenotypic features of 22q11.2DS and phen22q11.2 to guide optimal diagnostic approaches. We assessed 336 consecutive pediatric patients from three centers presenting with clin22q11.2 according to Tobias criteria. Diagnostic testing included fluorescence in situ hybridization or multiplex ligation-dependent probe amplification in all patients. In subsets of patients, additional investigations were performed as clinically indicated, including but not limited to karyotyping, chromosomal microarray analysis, and/or exome sequencing (ES) with CNV detection. To identify phenotypic differences, Fisher’s exact test and Chi-squared test were performed. Genetic abnormalities were identified in 127 patients, including 88 patients diagnosed with 22q11.2DS. Phen22q11.2 was identified in 39 patients, including *de novo* variants in 12 patients. Several SNVs were detected, including variants in recurrently affected genes, such as *CHD7* (*n* = 4), *TBX1* (*n* = 2), *JAG1* (*n* = 2), as well as variants in genes implicated in rare and ultra-rare diseases. We also described several rare and previously unreported clinical features associated with variants linked to phen22q11.2. No statistically significant phenotypic differences were observed between patients with phen22q11.2 and those with 22q11.2DS.

**Conclusions:**

Phen22q11.2 is genetically and phenotypically heterogeneous. The results support the use of ES with CNV analysis as a first-tier, high-throughput diagnostic approach in clin22q11.2, as comprehensive genomic testing is essential for improving diagnostic accuracy and optimizing both genetic counseling and clinical management in this population.

**Supplementary Information:**

The online version contains supplementary material available at 10.1186/s40348-026-00236-1.

## Background

The 22q11.2 deletion syndrome (22q11.2DS) is one of the most common microdeletion syndromes with an estimated incidence of 1 in 3000–6000 live births [1]. The phenotype associated with 22q11.2DS encompasses the clinical entities that were previously described as DiGeorge syndrome, velocardiofacial syndrome, conotruncal anomaly face syndrome, Opitz GBBB syndrome, and Cayler cardiofacial syndrome [1, 2].

Molecular diagnosis of 22q11.2DS is primarily based on the detection of the typical deletion in the 22q11.2 cytogenetic region using fluorescence in situ hybridization (FISH) or multiplex ligation-dependent probe amplification (MLPA), with deletion sizes ranging from 1.5 to 3.0 Mb [3].

Early recognition of 22q11.2DS is essential given the wide range of medical, neurodevelopmental, and hereditary consequences associated with this condition [1]. In practice, clinical diagnosis is particularly challenging owing to its heterogeneous presentation and the limited assessability of several features in early childhood [1]. Furthermore, due to variable severity and overlap with other rare disorders, diagnosis is frequently delayed [1].

Several studies have aimed to establish diagnostic criteria for clinically suspected 22q11.2DS (clin22q11.2); however, no consensus has been reached [4–6]. Tobias et al. proposed a criteria encompassing a broad spectrum of clinical, laboratory, and radiologic findings [4]. According to these criteria, core features include conotruncal cardiac anomalies, characteristic facial features, developmental delay, velopharyngeal insufficiency, cleft palate, hypocalcemia, thymus hypoplasia and immunodeficiency [4]. Additional symptoms consist of short stature, hypotonia, renal abnormalities, and psychiatric disorders [4]. Monteiro et al. later suggested a similar set of criteria with some modifications (e.g., reclassifying neurocognitive dysfunction as an associated feature and excluding short stature) [6]. It should be noted, however, that in practice, these criteria fail to identify nearly one-third of affected individuals [5].

In the current diagnostic landscape, the genetic diagnosis remains unclear for up to 40% of patients with clin22q11.2, which may, at least in part, be explained by other causative variants with overlapping phenotypes, hereafter referred to as phenocopies of 22q11.2DS (phen22q11.2) [7]. Phen22q11.2 generally includes CHARGE syndrome (*CHD7* variants), Smith-Lemli-Opitz syndrome (*DHCR7* variants), and Alagille syndrome (*JAG1* and *NOTCH2* variants) [8]. Additionally, other pathogenic copy number variants (CNVs) involving 10p13-p14, 11q23-ter, 4q35, 2p11.2, 8p23, and 17p13.3 regions should be considered [8–11].

Although previous studies have reported CNVs as phen22q11.2 [10, 12, 13], large-scale systematic screening for other causative variants, such as single nucleotide variants (SNVs), has not been performed. Therefore, we aim to investigate genetic variants associated with phen22q11.2 in order to accomplish definitive genetic diagnosis, reduce diagnostic delay, and provide accurate clinical management. Furthermore, to guide the selection of the most appropriate diagnostic approach in the clin22q11.2 cohort, we sought to investigate phenotypic differences between patients with 22q11.2DS and those with phen22q11.2.

## Methods

### Patients

In the first, retrospective phase of this ambidirectional cohort study, a total of 336 pediatric patients with clin22q11.2 were identified at three main pediatric centers in Budapest, who fulfilled the Tobias criteria for clin22q11.2 (*Supplementary Materials Table 1*) and were referred for genetic evaluation between 2005 and 2023. The term clin22q11.2 was used for individuals with a phenotype resembling 22q11.2DS in whom no genetic diagnosis had yet been established.

**Fig. 1:**
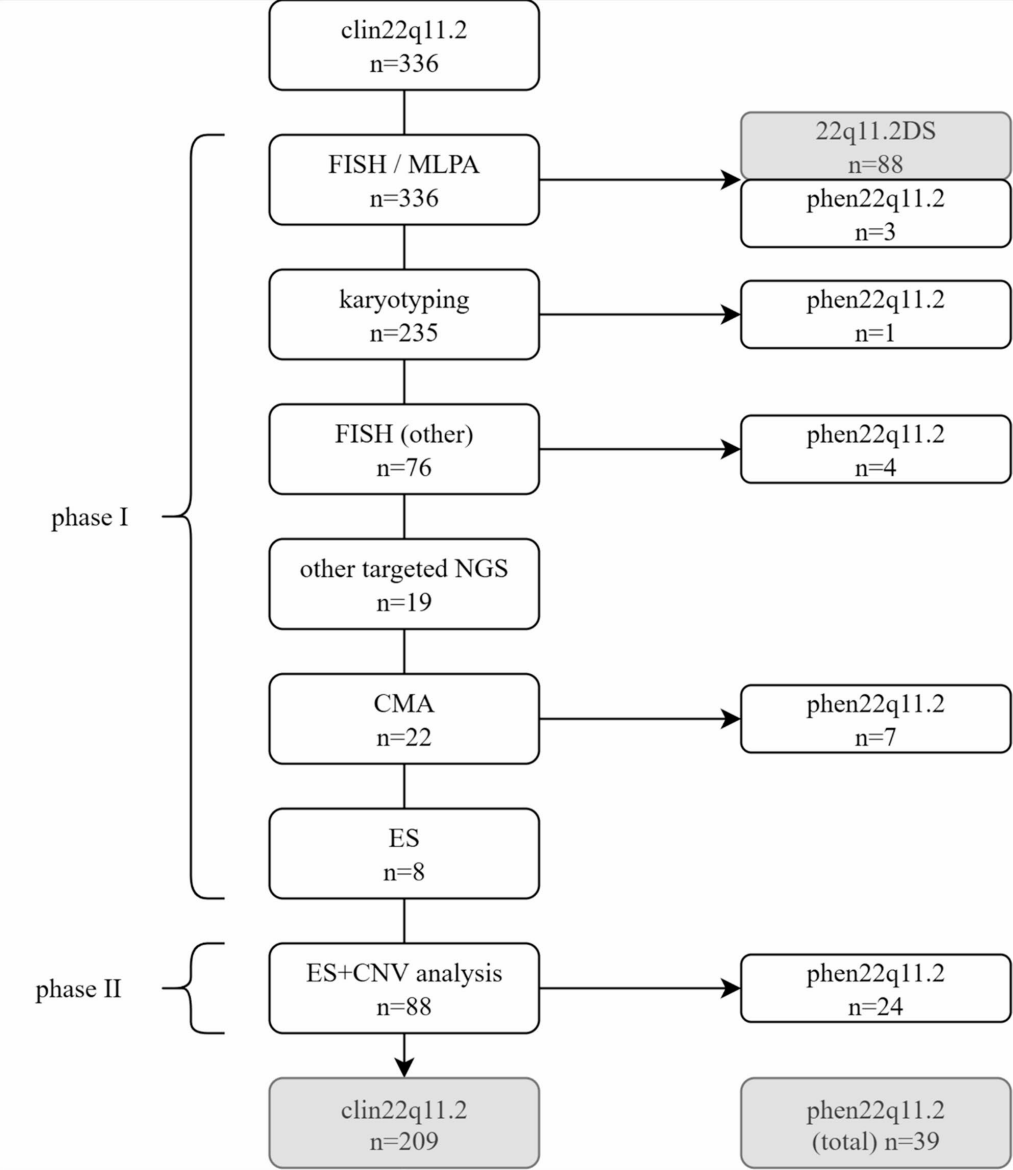
Overview of identified variants in the cohort. In the first, retrospective phase of this ambidirectional cohort study, a total of 336 pediatric patients with clin22q11.2 were identified. Clin22q11.2 patients in whom a causative variant other than the typical 22q11.2 deletion was identified were reclassified as phen22q11.2. Initial testing for 22q11.2DS using FISH and/or MLPA was performed in all 336 individuals with clin22q11.2, confirming the typical 22q11.2 deletion in 88 patients and identifying three with phen22q11.2. For individuals in whom the typical 22q11.2 deletion was not detected, additional genetic analyses were undertaken according to clinical presentation and within the constraints of the national healthcare funding system. Conventional karyotyping was carried out in 235 patients and detected one with phen22q11.2. Targeted FISH analyses at other loci were performed in 76 patients, yielding four diagnoses of phen22q11.2. Targeted NGS approaches were applied in 19 patients, without identifying any with phen22q11.2. CMA was conducted in 22 patients and identified seven with phen22q11.2. ES without CNV analysis was performed in eight patients and did not detect any with phen22q11.2. In phase II, patients were prospectively enrolled for ES with CNV analysis based on modified Tobias criteria. This approach was applied to 88 individuals and resulted in the identification of 24 patients with phen22q11.2. At the end of the study, a genetic diagnosis was established in 127 patients; however, no causative variant was identified in 209 patients with clin22q11.2. 22q11.2DS: 22q11.2 deletion syndrome, clin22q11.2: Clinically suspected 22q11.2 deletion syndrome, CMA: Chromosomal microarray analysis, CNV: Copy number variant, ES: Exome sequencing, FISH: Fluorescence in situ hybridization, MLPA: Multiplex ligation-dependent probe amplification, NGS: Next-generation sequencing, phen22q11.2: Phenocopies of 22q11.2 deletion syndrome, SNV: Single nucleotide variant

After the initial genetic testing for the identification of 22q11.2 deletion by FISH or MLPA, patients who were found not to harbor the deletion underwent subsequent genetic investigations that varied among patients (Fig. 1, *Supplementary Materials Table 2*). The selection of tests was determined by the clinical presentation and was constrained by the resources available within the national healthcare funding system. Patients with clin22q11.2 in whom a causative variant other than the typical 22q11.2 deletion was identified were reclassified as having phen22q11.2.

In the second phase of the study, patients were prospectively recruited for exome sequencing (ES) with CNV analysis (Fig. 1). Inclusion was restricted to patients with a phenotype considered to be more suggestive of 22q11.2DS. This was defined as fulfilling at least one criterion from column A or at least two criteria from column B according to the Tobias classification (*Supplementary Materials Table 1*) [4]. The presence of a milder phenotype (only one feature from column B together with one feature from column C) was considered insufficient for inclusion.

Individuals who had received a molecular diagnosis of 22q11.2DS or phen22q11.2 during phase I (*n* = 103, Fig. 1) were excluded from further recruitment. Additional exclusions included patients who could not be enrolled due to death, ongoing genetic examination, loss to follow-up, lack of consent, unavailable contact information, transition to adulthood, or other logistical reasons (*n* = 102).

In total, 88 pediatric patients were successfully recruited for ES with CNV analysis.

Phenotype was classified according to the Human Phenotype Ontology by board-certified clinical geneticists [14]. None of the families were known to be consanguineous. Patients and their legal representatives provided written informed consent (ETT-TUKEB IV/8613-1/2021/EKU).

### Genetic analysis

#### Karyotyping, fluorescence in situ hybridization and multiplex ligation-dependent probe amplification

Conventional G-banded karyotyping was performed on peripheral blood samples using standard diagnostic techniques. FISH analysis for the 22q11.2 deletion was performed using commercially available DiGeorge TUPLE1 and N25 probes (Cytocell, Cambridge, UK) according to the manufacturer’s protocols. If required, additional FISH analyses were performed according to the manufacturer’s protocols. The list of FISH probes targeting other loci is provided in *Supplementary Materials Table 3*. MLPA was performed according to the manufacturer’s specifications (MRC Holland, Amsterdam, Netherlands) using SALSA MLPA Probemix P250 kit (MRC Holland, Amsterdam, Netherlands).

#### Targeted next-generation sequencing approaches

In some patients, additional targeted gene panel sequencing was performed at external institutions; therefore, detailed methodological information was not available.

#### Chromosomal microarray analysis

The platforms and analysis software used for chromosomal microarray analysis (CMA) were NimbleGen Array (CGX 1.4 M) with NimbleGen MS 200 Microarray Scanner (Roche NimbleGen Inc., Madison, WI, USA) and Affymetrix CytoScan 750 K with Affymetrix Genechip Scanner and Chromosome Suite Analysis (ChAS) 4.0 (Thermo Fisher Scientific, Inc.; Waltham, MA, USA).

#### Exome sequencing

Exome capture was performed using xGen Exome Research Panel v2, supplemented with xGen human mtDNA panel and xGen Custom Hyb Panel v1 (Integrated DNA Technologies, Coralville, Iowa, USA). Sequencing was performed using NovaSeq 6000 (Illumina, San Diego, CA, USA). Variant interpretation was performed using EVIDENCE, based on the guidelines recommended by the American College of Medical Genetics and Genomics [15]. Reads were mapped against the human GRCh38/hg38 reference genome.

#### Variant validation and family segregation studies

All variants of interest identified by ES were validated, SNVs by Sanger sequencing, CNVs by FISH and G-banded karyotyping as described in "Karyotyping, fluorescence in situ hybridization and multiplex ligation-dependent probe amplification" section. In the case of a 4 kb deletion at Xp11.4, quantitative multiplex PCR of short fluorescent fragments was performed as described previously [16]. Primers are listed in *Supplementary Materials Tables 4–5.*

### Statistical analysis

The statistical analysis was applied to investigate whether particular clinical features might be associated with a genotype-positive or genotype-elusive group.

Therefore, patients were divided into four groups: patients with 22q11.2DS (Group A); patients with phen22q11.2 caused by CNVs (Group B); patients with phen22q11.2 caused by SNVs (Group C); and patients who underwent ES with CNV analysis or both ES and CMA, but for whom no causative variant was identified (Group D). The selection of Group D was based on the requirement that both SNVs and CNVs had been systematically assessed.

Patients with 22q11.2DS and an additional CNV or SNV outside the 22q11.2 cytogenetic region in Group A (*n* = 5), CNVs involving the 22q11.2 region in Group B (*n* = 3), SNVs associated only with isolated features in Group C (*n* = 1), and variants of unknown significance (VUSs, *n* = 5) in Group D were excluded from analysis.

Statistical analysis was performed using Python (v3.13.2; Python Software Foundation, Wilmington, DE, USA). Phenotypic features of groups were compared using Fisher’s exact test and Chi-squared test with statistical significance set at *p* < 0.05. Pairwise comparisons were performed between all groups. Post-hoc comparisons were adjusted using the Bonferroni correction.

The figure was created using draw.io online diagram software.

## Results

### Genetic test results

Causative variants were identified in 127 patients: FISH and MLPA detected 22q11.2DS in 88 patients (26.19%), as summarized in Fig. 1. Phen22q11.2 was established in 39 patients through additional genetic testing, including causative CNVs in 21 patients and causative SNVs in 18 patients (Fig. 1; Table 1).

**Table 1:** Clinical and genetic characteristics of patients with phen22q11.2

I. CNVs						
Patient ID	Molecular locus	Critical genes	CNV size	Seg	Clinical interpretation	Phenotype
DS21	arr[GRCh38]22q11.21(18929329_21562851)x3	*TBX1*,* DGCR8*,* CRKL*, *GP1BB*,* SNAP29*,* SCARF2*,* COMT*,* PAX1*,* TANGO2*	2634 kb	ND	22q11.2 duplication syndrome (MIM:608363)	6-year-old girl with GDD, behavioural disorder, myopia, strabismus, widely spaced tooth, low-set ears, broad nasal bridge, high and narrow palate, loose joints, bilateral fifth finger clinodactyly
DS22	arr[GRCh38]22q11.22(21946280_22218088)x3	*PPM1F*,* TOP3B*	272 kb	ND	22q11.2 duplication syndrome (MIM:608363)	3-year-old girl with cleft lip and palate, GDD, learning difficulties, short stature, long philtrum, high and narrow palate, prominent forehead
DS23	arr[GRCh38]22q11.21q11.22(21106351_22620492)x1	*MAPK1*	1502 Mb	ND	Distal type 22q11.2 deletion syndrome (MIM :611867)	10-year-old boy with ASD, GDD, microcephaly, cleft palate, recurrent otitis media, hearing loss, short fingers, wide and depressed nasal bridge, umbilical hernia, hypertelorism, epicanthus, broad nasal bridge
DS24	rsa10p14(P250)(8,058,591 − 11,165,627)x1	*GATA3*,* TCEB1P3*,* CELF2*	ND	ND	DiGeorge syndrome Type II (MIM:601362)	3-year-old boy with ASD, persistent superior vena cava, muscle hypotonia, GDD, ID, bilateral hearing loss, hypoparathyroidism, VUR, hypoglobulinemia, recurrent infections, thrombocytopenia, inguinal hernia, hypertelorism, broad and depressed nasal bridge, high and narrow palate, retrognathia, low-set ears, clinodactyly of the fourth toe
DS25	NC_000010.10:g.79937852_87358431del	*BMPR1A*	7.42 Mb	*dn*	Chromosome 10q22.3-q23.2 deletion syndrome (MIM:612242)	7-year-old boy with TOF, delayed speech and language development, axial hypotonia, recurrent infections, renal hypoplasia, scoliosis, asthma
DS26	NC_000018.9:g.65762843_80160606del NC_000020.10:g.96005_19722926dup	ND	14.49 Mb 19.62 Mb	pat**	Chromosome 18q deletion syndrome (MIM: 601808) Trisomy 20p syndrome (ORPHA:261318)	12-year-old boy with ASD, VSD, GDD, bilateral hearing impairment, hypoplastic hippocampus, muscle hypotrophy, nystagmus, axial hypotonia, recurrent infections, cleft hard and soft palate, thoracolumbar kyphosis, bilateral Inguinal hernia, umbilical hernia and minor anomalies (asymmetric ears, protruding ears, hypertelorism, ulnar deviation of thumb, left single transverse palmar crease, thin upper lip vermilion, broad forehead, bilateral II-III overlapping toe, wide and depressed nasal bridge)
DS27	arr[GRCh38]3q26.1q29(161581071_198124573)x3; arr[GRCh38]18p11.32p11.31(136226_7089933)x1;	ND	36.5 Mb 6.95 Mb	mat**	ND Chromosome 18p deletion syndrome (MIM 146390)	2-year-old boy with VSD, GDD, focal epilepsy, cleft palate, broad nasal bridge, upturned nasal tip, prominent forehead, low-set ears, wide mouth, thick lips, crumpled auricles, hypospadias, duplex kidneys, hypoglobulinemia
DS28	46,XX, del(18)(p11.2)	*HPE4*	ND	ND	Chromosome 18p deletion syndrome (MIM 146390)	9-year-old girl with GDD, broad depressed nasal bridge, prominent forehead, muscle hypotonia, ID, hypothyroidism, obesity, VPI
DS29	NC_000001.10:g.147687440_148456457del	*GJA8*,* GJA*,* BCL9*	769.5Kb	ND	Chromosome 1q21.1 deletion syndrome (MIM:612474)	3-year-old boy with AVSD, GDD, muscle hypotrophy, hippocampal malrotation, generalized hypotonia, decreased circulating IgG and IgA level, strabismus, joint hypermobility and minor anomalies (hypertelorism, micrognathia, short philtrum, low-set ears)
DS30	arr[GRCh38]1p36.33p36.22(884621_9287941)x1	*GABRD*,* KCNAB2*,* SKI*,* RERE*,* UBE4B*,* GNB1*,* PRDM16*	8.4 Mb	ND	Chromosome 1p36 deletion syndrome (MIM:607872)	3-year-old girl with TOF, epilepsy, hirsutism, muscle hypotonia, stereotypical tongue movements, clapping, West syndrome, minor anomalies
DS31	NC_000002.11:g.239961118_241182823del	ND	1.2 Mb	*dn*	Chromosome 2q37 deletion syndrome (MIM:600430)	11-year-old girl with ASD, GDD, generalized hypotonia, attention deficit disorder, recurrent infections, horseshoe kidney, anal atresia, short stature, laryngomalacia, asthma and minor anomalies (hypotelorism, micrognathia, smooth philtrum, low-set ears, hypertrichosis, long fingers, pectus excavatum, microcephaly)
DS32	NC_000004.11:g.124671_2378027dup NC_000008.10:g.435615_6732650del	*GATA4*,* SOX7*, *NEIL2*,* DLGAP2*, *CLN8*,* ARHGEF10*	23.7 Mb 6.2 Mb	pat**	4p16.3p15.2 duplication 8p23.3p23.1 deletion (ORPHA:251071)	6-year-old girl with ASD, VSD, GDD, astigmatism, hypermetropia and minor anomalies (straight eyebrow, long and smooth philtrum, thin upper lip vermilion)
DS33	rsa4q35.1,4q35.2(P250)(186,303,263 − 187,390,323)x1	*SLC25A4*,* KLKB1*	ND	ND	Distal 4q deletion syndrome (ORPHA:96145)	6-year-old girl with VSD, persistent left superior vena cava, GDD, generalized hypotonia, VUR, scoliosis, duplex kidneys, recurrent infections, broad nasal bridge, long philtrum, thin upper lip, anteverted nostrils, macroglossia, low-set ears, umbilical hernia
DS34	47,XX,+8[14]/46,XX[2] nuc ish(D8Z1)x3[93]/(D8Z1)x2[7]	ND	ND	ND	Trisomy 8 mosaicism syndrome (ORPHA:96061)	2-year-old girl with GDD, hypertelorism, low-set ears, broad forehead, agenesis of the corpus callosum, ventricular enlargement
DS35	NC_000023.10:g.41168007_41171958del	ND	4Kb	mat (mosaic)	X-linked intellectual developmental disorder (MIM:300968)	8-year-old girl with ASD, VSD, hypoplasia of the corpus callosum, generalized hypotonia, recurrent infections, dilated renal pelvis, anteriorly placed anus and minor anomalies (wide nasal bridge, high, narrow palate, micrognathia, low-set ears, facial asymmetry)
DS36	nuc ish(DXZ1)x1[25]/(DXZ1)x2[75]	*SHOX*	ND	ND	Mosaic Turner syndrome (ORPHA:881)	1-year-old girl with IAA-B, thymus hypoplasia, extreme connective tissue laxity
DS37	46,XX, t(6;15)(q31;q26) arr[GRCh38]13q33.3(106788118_108387927)x1	*LIG4*	N/D 1.6 Mb	*dn*	Balanced reciprocal translocation involving 6q31 and 15q26 13q33-q34 deletion syndrome (MIM :619148)	3-year-old girl with GDD, muscle hypotonia, epicanthus, long philtrum, thick lower lip, loose finger joints, clinodactyly of the second toe, scoliosis
DS38	arr[GRCh38]9p24.3q34.3(208454_138124196)x3[0.67]	ND	137,916 kb	ND	Mosaic 9 trisomy syndrome (ORPHA:99776)	1-year-old girl with ASD, VSD, delayed motor development, muscle hypotonia, micrognathia and retrognathia, low-set ears, atopic dermatitis, dislocated hips, pyelectasia
DS39	rsa9q34.3(P250)(137,618,992 − 137,870,016)x1	*EHMT1*	ND	ND	Kleefstra syndrome (MIM:610253)	2-year-old boy with TA, GDD, severe infections, thymus hypoplasia, lymphocytopenia, hypocalcemia, hypertelorism, anteverted nares, broad and depressed nasal bridge, micrognathia and retrognathia, thick upper lip, curved mouth corners, low-set ears, microcephaly, left main bronchus stenosis
DS40	46,XX, del(9)(q34)[2]/46,XX[13] nuc ish(ABL1)x1[8]/(ABL1)x2[92]	*EHMT1*	ND	*dn*	Mosaic Kleefstra syndrome (MIM:610253)	1-year-old girl with VSD, cleft palate, low-set ears, micrognathia, delayed psychomotor development, feeding difficulties, and VPI
DS41	46,XY, del(7)del(7)(p11.2-p14) ish(subtel7p, subtel7q)x2	ND	ND	ND	7p interstitial deletion	1-year-old boy with ASD, VSD, hippocampal malrotation, delayed myelination, corpus callosum hypoplasia, horseshoe kidney, long and flat philtrum, inguinal hernia, polydactyly, flat forehead, epicanthus, broad and depressed nasal bridge

II. SNVs						
Patient ID	Gene	Nucleotide change	Zygosity	Seg.	Related disorder	Phenotype
**DS1**	***TBX1***	**NM_001379200.1:c.438–2 A > T**	het	pat	DiGeorge syndrome (MIM:188400)	4-year-old girl with IAA-B, thymus aplasia, hypoparathyroidism, and minor anomalies (low-set ears, low anterior hairline, short neck and limbs)
**DS2**	***TBX1***	**NM_001379200.1:c.612 C > G(p.Tyr204Ter)**	het	mat	DiGeorge syndrome (MIM:188400)	9-year-old girl with pulmonary atresia, VSD, MAPCA, ARSA, bilateral sensorineural hearing impairment, GDD and minor anomalies (micrognathia, retrognathia, epicanthus)
DS3	*CHD7*	NM_017780.4:c.6157 C > T(p.Arg2053Ter)	het	*dn*	CHARGE syndrome (MIM:214800)	2-year-old girl with ASD, pulmonary stenosis, GDD, left sensorineural hearing impairment, hyperparathyroidism, coloboma and minor anomalies (epicanthus, anteverted nares, high and narrow palate, retrognathia, low-set ears, bilateral single transverse palmar crease, overlapping toes, hypertelorism)
**DS4**	***CHD7***	**NM_017780.4:c.3327G > C(p.Arg1109Ser)**	het	*dn*	CHARGE syndrome (MIM:214800)	9-year-old boy with TOF, RAA, MAPCAs, ARSA, developmental delay, ADHD, brain stem hypoplasia, asthma and minor anomalies (large ears, microcephaly, overlapping toes)
**DS5**	***CHD7***	**NM_017780.4:c.3310_3311del(p.Ile1104Ter)**	het	ND*	CHARGE syndrome (MIM:214800)	9-year-old girl with TOF and severe pulmonary stenosis, thymus hyperplasia, GDD, ID, axial hypotonia, bilateral hearing impairment, coloboma, microphthalmia, hypocalcemia, central precocious puberty, asthma and minor anomalies (high and narrow palate, large ears, microcephaly)
**DS6**	***CHD7***	**NM_017780.4:c.4505del(p.Ser1502Ter)**	het	*dn*	CHARGE syndrome (MIM:214800)	8-year-old girl with TOF, RAA, bilateral hearing impairment, BPPV, GDD, coloboma and short stature
DS7	*JAG1*	NM_000214.3:c.550 C > T(p.Arg184Cys)	het	*dn*	Alagille syndrome (MIM:118450)	6-year-old girl with TOF, thymus hypoplasia, hypercalcemia, hydronephrosis and minor anomalies (wide and depressed nasal bridge, high and narrow palate, micrognathia, short philtrum, pectus excavatum)
**DS8**	***JAG1***	**NM_000214.3:c.1080dup(p.Glu361Ter)**	het	pat	Alagille syndrome (MIM:118450)	4-year-old boy with TOF, pulmonary valve agenesis, lymphocytopenia, severe recurrent infections and minor anomalies (hypertelorism, wide nasal bridge, pectus excavatum)
DS9	*NOTCH1*	NM_017617.5:c.599G > T(p.Gly200Val)	het	pat	Aortic valve disease (MIM:109730)	11-year-old girl with TOF, non-confluent pulmonary arteries, RAA, ARSA, GDD, attention deficit disorder, generalized hypotonia, recurrent infections and minor anomalies (micrognathia, retrognathia)
DS10	*TMEM260* *TMEM260*	NM_017799.4:c.721dup(p.Tyr241LeufsTer3) NM_017799.4:c.942-10T > C	comp. het	trans	Structural heart defects and renal anomalies syndrome (MIM:617478)	18-year-old girl with TOF, agenesis of the pulmonary vessels, MAPCA, generalized hypotonia, microcephaly and minor anomalies (hypertelorism, long philtrum, thick lower lip vermilion, synophrys)
DS12	*NR2F2*	NM_021005.4:c.103_109del(p.Gly35ArgfsTer75)	het	ND	46,XX sex reversal 5 (MIM:618901)	17-year-old boy with ASD, VSD, GDD, ADHD, autism spectrum disorder, hypopituitarism, multicystic kidney dysplasia, unilateral renal agenesis, myopia, nasal speech and minor anomalies (low-set ears, retrognathia, long and smooth philtrum)
**DS14**	***PHF8***	**NM_015107.3:c.718 C > T(p.Arg240Ter)**	hem	ND	X-linked intellectual developmental disorder (MIM:300263)	12-year-old boy with TOF, RAA, low levels of circulating IgM and IgG, generalized hypotonia, GDD, ID, attention deficit, bilateral hearing impairment, demyelination, renal hypoplasia, VUR, umbilical hernia, nasal speech and minor anomalies (thin upper lip vermilion, high and narrow palate, supernumerary teeth, retrognathia, pectus excavatum, bilateral transverse palmar crease and long toes)
**DS15**	***ELN***	**NM_000501.4:c.1055_1056del** **(p.Pro352ArgfsTer23)**	het	mat	Supravalvular aortic stenosis (MIM:185500)	2-year-old boy with supravalvular aortic stenosis, supravalvular pulmonary stenosis, generalized hypotonia, hypocalcemia and minor anomalies (wide nasal bridge, epicanthus, thick lower lip vermilion, high and narrow palate, long and thick eyelashes)
**DS16**	***CDK13***	**NM_003718.5:c.382 C > T(p.Gln128Ter)**	het	ND	Congenital heart defects, dysmorphic facial features, and intellectual developmental disorder (MIM:617360)	5-year-old boy with TA, ASD, VSD, GDD, hypothyroidism, hyponatremia, scoliosis, severe neonatal feeding difficulty and minor anomalies (thick eyelashes, wide and depressed nasal bridge, low-set ears and flat occiput)
**DS17**	***CNOT1***	**NM_016284.5:c.439 C > T(p.Gln147Ter**)	het	*dn*	Vissers-Bodmer syndrome (MIM:619033)	6-year-old boy with GDD, behavioural disorder, attention deficit, generalized hypotonia, recurrent infections, short stature, asthma and minor anomalies (low-set ears, depressed nasal bridge, thin upper lip vermilion, micrognathia, retrognathia, short philtrum, toe clinodactyly)
**DS18**	***ZEB2***	**NM_014795.4:c.656dup(p.Tyr220LeufsTer19)**	het	*dn*	Mowat-Wilson syndrome (MIM:235730)	1-year-old girl with ASD, corpus callosum agenesis, GDD, ID, hydronephrosis, hydroureter, Hirschsprung’s disease
**DS19**	***MED13L***	**NM_015335.5:c.3401G > A(p.Cys1134Tyr)**	het	*dn*	Impaired intellectual development and distinctive facial features with or without cardiac defects (MIM:616789)	8-year-old girl with ASD, GDD, epilepsy, generalized hypotonia, cleft palate, short neck, clubfoot, scoliosis, arthrogryposis congenita, ventriculomegaly, cerebral cyst, narrowed auricular canals and minor anomalies (upslanted palpebral fissure, microretrognathia, low-set ears, prominent forehead, low anterior hairline, microcephaly)
DS20	*SON*	NM_138927.4:c.5753_5756del(p.Val1918GlufsTer87)	het	*dn*	ZTTK syndrome (MIM:617140)	12-year-old girl with ASD, VSD, GDD, ataxia, attention deficit disorder, corpus callosum agenesis, generalized hypotonia, dyscrania, strabismus, left inguinal hernia and minor anomalies (hypertelorism, high and narrow palate, low-set ears, dolichocephaly, broad forehead, epicanthus)

Novel variants are shown in bold

*ADHD* Attention deficit hyperactivity disorder, *ARSA* Aberrant right subclavian artery, *ASD* Atrial septal defect, *AVSD* Atrioventricular septal defect, *BPPV* Benign paroxysmal positional vertigo, *CNV* Copy number variant, *comp. het* Compound heterozygous, *dn de novo*, *GDD* Global developmental delay, *het* Heterozygous, *hem* Hemizygous, *IAA-B* Interrupted aortic arch type B, *ID* Intellectual disability, *MAPCA* Major aortopulmonary collateral arteries, *mat* Maternal, *ND* No data, *nuc ish* Nucleal in situ hibridization, *pat* Paternal, *phen22q11.2* Phenocopies of 22q11.2 deletion syndrome, *RAA* Right aortic arch, *rsa* Region specific array, *SNV* Single nucleotide variant, *TA* Truncus arteriosus, *TOF* Tetralogy of Fallot, *VPI* Velopharyngeal insufficiency, *VSD* Ventricular septal defect, *VUR* Vesicoureteral reflux, *: not identified in the mother, **: balanced translocation in the parent

#### The identified CNVs

CMA detected three additional variants within the 22q11.2 region in three patients: two 22q11.2 duplications (DS21, DS22) -one of which (DS21) is reciprocal to the typical deletion- and one distal 22q11.2 deletion (DS23, LCR22 D-F) (Table 1). Heterogeneous CNVs outside the 22q11.2 region were identified in 18 patients (Table 1). Three patients (DS26, DS27, DS32) harbored multiple CNVs resulting from parental balanced translocations, and one patient (DS37) had a 13q33.3 deletion together with a balanced translocation (Table 1). *De novo* CNVs were found in four patients.

#### The identified SNVs

ES identified pathogenic and likely pathogenic SNVs in 18 patients, including 12 novel variants (shown in bold in Table 1). Supporting evidence for the pathogenicity of these variants is presented in Table 2. *De novo* SNVs were identified in 8/14 patients. Only three genes were found to be affected in multiple patients: *CHD7* (*n* = 4), *TBX1* and *JAG1* (each in two patients) (Table 1)*.* Moreover, we identified rare and ultra-rare disorders within the cohort, including *CNOT1*-associated Vissers-Bodmer syndrome (DS17), *ZEB2*-associated Mowat-Wilson syndrome (DS18), *SON*-associated Zhu-Tokita-Takenouchi–Kim syndrome (DS20), and *TMEM260*-associated Structural Heart Defects and Renal Anomalies syndrome (DS10) (Table 1).

**Table 2:** Evidence supporting the pathogenicity of novel variants

Patient ID	Gene	Nucleotide change	Evidence of pathogenicity	Seg.	ACMG	Phenotype overlap
DS1	*TBX1*	NM_001379200.1: c.438–2 A > T	PVS1, PM2	pat	LP	yes
DS2	*TBX1*	NM_001379200.1: c.612 C > G (p.Tyr204Ter)	PVS1, PM2	mat	LP	yes
DS4	*CHD7*	NM_017780.4: c.3327G > C (p.Arg1109Ser)	PM2, PP2, PP3	*dn*	LP	yes
DS5	*CHD7*	NM_017780.4: c.3310_3311del (p.Ile1104Ter)	PVS1, PM2	ND	LP	yes
DS6	*CHD7*	NM_017780.4: c.4505del (p.Ser1502Ter)	PVS1, PM2	*dn*	LP	yes
DS8	*JAG1*	NM_000214.3: c.1080dup (p.Glu361Ter)	PVS1, PM2	pat	LP	yes
DS14	*PHF8*	NM_015107.3: c.718 C > T (p.Arg240Ter)	PVS1, PM2	ND	LP	yes
DS15	*ELN*	NM_000501.4: c.1055_1056del (p.Pro352ArgfsTer23)	PVS1, PM2	mat	LP	only partial
DS16	*CDK13*	NM_003718.5: c.382 C > T (p.Gln128Ter)	PVS1, PM2	ND	LP	yes
DS17	*CNOT1*	NM_016284.5: c.439 C > T (p.Gln147Ter)	PVS1, PM2	*dn*	LP	yes
DS18	*ZEB2*	NM_014795.4: c.656dup (p.Tyr220LeufsTer19)	PVS1, PM2	*dn*	LP	yes
DS19	*MED13L*	NM_015335.5: c.3401G > A (p.Cys1134Tyr)	PM2, PP2, PP3	*dn*	LP	yes

The table summarizes SNVs identified in patients with clin22q11.2, together with the associated genes, nucleotide changes, and evidence supporting pathogenicity according to ACMG criteria. Most variants were classified based on criteria such as PVS1 (null variant in a gene where loss of function is a known mechanism), PM2 (absent from controls), PP2 (missense variant in a gene with a low rate of benign variation), and PP3 (computational evidence supporting a deleterious effect). Parentally inherited variants were accepted as disease-causing only if supporting literature data regarding decreased prevalence were available

*ACMG* American College of Medical Genetics and Genomics, *clin22q11.2* Clinically suspected 22q11.2 deletion syndrome, *dn de novo*, *mat* Maternal, *LP* Likely pathogenic, *ND* No data, *pat* Paternal, *SNV* Single nucleotide variant

The overall diagnostic yield for ES with CNV analysis was 27.27% (24/88 patients).

#### Unreported and rare phenotypic features

Several rare or previously unreported clinical features were identified (Table 1). These include cleft palate and arthrogryposis in association with *MED13L* (DS19); attention deficit hyperactivity disorder and hypopituitarism with *NR2F2* (DS12); ventricular septal defect with *SON* (DS20); tetralogy of Fallot and microcephaly with *TMEM260* (DS10); precocious puberty and hypoparathyroidism with *CHD7* (DS5); truncus arteriosus and hypothyroidism with *CDK13* (DS16); and congenital heart disease (CHD), urinary tract malformations and hypogammaglobulinemia with *PHF8* (DS14) variants (Table 1).

##### Variants associated with congenital heart disease

Variants in *CDK13* are typically associated with septal defects, intellectual disability (ID), global developmental delay (GDD), and characteristic facial features [17]. Patient DS16, however, presented with truncus arteriosus alongside clinical features such as hyponatremia and hypothyroidism (Table 1), which have not previously been associated with this gene [17]. Similarly, *PHF8*-related disorders are primarily characterized by craniofacial anomalies, GDD, and behavioral issues, all of which were present in patient DS14 (Table 1) [18]. Notably, this patient also exhibited conotruncal CHDs, urinary tract malformations, and hypogammaglobulinemia -features not previously reported in this disorder [18].

##### Variants associated with immunodysfunction

Core features of CHARGE syndrome include CHD, GDD, hearing loss, coloboma, choanal atresia, genitourinary anomalies, and possible immune dysfunction [19]. The overlapping phenotype may be due to shared defects in neural crest and pharyngeal arch development, as seen in CHARGE syndrome, which complicates the clinical distinction between the two syndromes [20]. Patient DS5, however, exhibited thymic hyperplasia and precocious puberty, in contrast to the delayed puberty more typically observed in this syndrome (Table 1) [21]. Similarly, *JAG1* variants traditionally linked to Alagille syndrome were identified in patients presenting with isolated cardiac defects or extrahepatic features [22, 23]. Previous studies have reported individuals with features of Alagille syndrome -including CHDs, minor anomalies, renal and immunodysfunction-but without liver involvement, as observed in patients DS7 and DS8 (Table 1) [23–25].

##### Variants associated with central nervous system and craniofacial abnormalities

*MED13L*-related disorder typically involves craniofacial anomalies, GDD, hypotonia, short stature, and CHD [26]. The presence of cleft palate and arthrogryposis in patient DS19 (Table 1), though rare, emphasizes the relevance of these features to the phenotype [26–30]. While *NOTCH1* variants are well-established in the pathogenesis of CHDs, they are increasingly associated with a broader clinical spectrum, including central nervous system, craniofacial, and limb anomalies [31, 32]. Although *NOTCH1* is traditionally examined in the context of Adams–Oliver syndrome, emerging evidence indicates that many affected individuals exhibit phenotypes that do not meet the diagnostic criteria, as seen in patient DS9 (Table 1) [31]. Patient DS12 harbored a causative variant in *NR2F2 *(Table 1), a gene associated with multiorgan congenital anomalies [33, 34]. While common features include GDD, CHDs, and minor anomalies, this patient also exhibited attention deficit hyperactivity disorder and hypopituitarism [33, 34].

### Phenotype comparison

Comparisons of clinical features across the four patient groups (Groups A-D) were performed (the main phenotypic features are shown in Table 3). No statistically significant phenotypic differences were observed between patients with phen22q11.2 and those with 22q11.2DS.

**Table 3:** Main clinical features across four patient groups used for statistical analysis

Clinical feature	Group A *n* = 83	Group B *n* = 18	Group C *n* = 17	Group D *n* = 61
Tetralogy of Fallot	20/81	3/17	9/17	20/61
Interrupted aortic arch	12/82	1/17	1/17	3/61
Truncus arteriosus	8/82	1/17	1/17	8/61
Major aorto-pulmonary collateral arteries	8/81	0/17	3/17	4/61
Atrial septal defect	4/82	2/17	3/17	8/61
Ventricular septal defect	18/82	7/17	1/17	15/61
Right aortic arch	18/82	0/17	4/17	10/61
Aberrant right subclavian artery	5/82	0/17	3/17	1/61
Hypertelorism	18/48	5/15	3/16	9/44
Broad nasal bridge	12/49	7/15	3/16	14/44
Micrognathia	20/48	6/15	5/16	10/44
Retrognathia	8/48	3/15	7/16	7/44
Low-set ears	26/48	10/15	7/16	10/44
High and narrow palate	15/48	2/15	5/16	11/44
Developmental delay	42/63	13/16	12/17	24/40
Delayed speech development	29/40	5/9	7/14	18/27
Intellectual disability	18/35	3/8	8/14	5/25
Cleft palate	6/70	3/17	1/17	6/41
Velopharyngeal inssuficiency	6/28	2/5	0/9	1/31
Hypocalcaemia	18/75	2/12	1/17	3/58
Recurrent infections	37/65	8/12	3/16	22/41
Lymphocytopenia	15/62	1/12	2/16	8/50
Thymus hypoplasia	11/32	0/4	2/15	1/25
Short stature	6/54	1/8	4/17	4/46
Hypotonia	20/50	10/15	7/17	16/43

The statistical analysis was applied to investigate whether particular clinical features might be associated with a genotype-positive or genotype-elusive group. To address this question, the phenotypes of four groups (Groups A-D) were analyzed

From the molecularly confirmed 22q11.2DS group (Group A), patients who had an additional independent molecular diagnosis in addition to the 22q11.2 deletion (*n* = 5) were excluded, resulting in 83 patients being included in this group. For patients with phen22q11.2 caused by CNVs (Group B), CNVs involving the 22q11.2 region itself were excluded (*n* = 3), resulting in 18 patients being included. For patients with phen22q11.2 caused by SNVs (Group C), one variant accounting for only a single symptom of the complex phenotype (*n* = 1) was excluded; thus, 17 patients were included in the analysis. Group D involved patients for whom both SNVs and CNVs were systematically assessed by ES with CNV analysis or by ES and CMA, but no causative variant was identified. Of the initial 66 patients, five were excluded due to the presence of VUSs, resulting in 61 patients being included in the final analysis

Values are presented as the number of affected patients over the number of evaluated patients

*22q11.2DS* 22q11.2 deletion, *CNV* Copy number variant, *CMA* Chromosomal microarray analysis, *ES* Exome sequencing, *phen22q11.2* Phenocopies of 22q11.2 deletion syndrome, *SNV* Single nucleotide variant

## Discussion

This study aimed to identify patients with phen22q11.2 and to explore potential phenotypic differences between those with 22q11.2DS and those with phen22q11.2.

### Identified variants in the clin22q11.2 cohort

#### CNVs

22q11.2DS was diagnosed in 88 patients (26.19%), which is in agreement with literature data [5]. Previously reported diagnostic rates ranged from 4% to 78.2%, reflecting considerable variability due to differences in study design and patient selection criteria [5]. As previously proposed, 22q11.2 duplication syndrome is likely underdiagnosed, potentially due to its reduced penetrance and the presence of milder or less distinct congenital anomalies compared to the more recognizable 22q11.2DS [35]. In addition to two patients diagnosed with Kleefstra syndrome, numerous rare and rarely reported CNVs were identified (Table 1). Several of these -such as deletions at 1p36, 1q21.1, 2q37.3, 10p14, 4q35.1, 8p23.3, 45,X, and duplication at 4p16.3- have been previously reported in association with phen22q11.2 [10, 12, 13, 36]. In contrast, other CNVs -including deletions at 18q22, 18p11.21, 10q22.3, Xp11.4, 13q33, and 7p11.2p14- have not been described in this context. Three patients (DS26, DS27, DS32) had multiple CNVs (Table 1), highlighting that the phenotype may be complex and may arise from multiple genomic alterations, such as coexisting CNVs [37].

#### SNVs

In addition to CNVs, 18 SNVs were identified in the cohort, including variants in recurrently affected genes (*CHD7*,* TBX1*,* JAG1*), which have previously been reported in association with phen22q11.2 [8, 38, 39], as well as genes implicated in rare or ultra-rare disorders (*CNOT1*,* ZEB2*,* SON*,* TMEM260*) (Table 1). Incomplete penetrance was considered in patients DS1 and DS2 carrying *TBX1* variants, as well as in DS8 and DS9 with variants in *JAG1* and *NOTCH1*, consistent with previous reports [40–43].

Within the patient cohort selected for ES with CNV analysis, the diagnostic yield was 20.45% for SNVs and 6.8% for CNVs, comparable to that reported in previous studies [44–46].

### Unreported and rare phenotypic features

Although it has been reported that rare pathogenic SNVs and indels in *TBX1* can result in a phenotype similar to that of 22q11.2DS [40, 41], little is known about the phenotype of other phen22q11.2-associated SNVs. We describe several rare or previously unreported clinical features associated with pathogenic variants linked to phen22q11.2 (Table 1). While these observations require further validation through functional studies and larger patient cohorts, they illustrate the potentially expanding clinical spectrum linked to these genes.

### Phenotype analysis

When comparing phenotypes between patients with 22q11.2DS and those with phen22q11.2, no statistically significant differences were observed. These findings highlight the complexity of the phenotype and the absence of distinctive clinical features that would reliably differentiate 22q11.2DS from phen22q11.2.

As our results emphasize, the clinical diagnosis of 22q11.2DS is challenging. One reason is the extremely broad phenotypic spectrum of the condition: more than 180 associated clinical features have been described [47]. It should be noted that symptom evaluation may be age dependent; therefore, as children grow and new symptoms emerge, reevaluation may be necessary [1]. Second, there are currently no universally accepted objective clinical diagnostic criteria that reliably distinguish 22q11.2DS from other conditions [4, 6]. As our results also suggest, even experienced specialists may find it difficult to establish an accurate clinical diagnosis before genetic testing. Third, many other genetic syndromes share overlapping phenotypic characteristics with 22q11.2DS, making differential diagnosis based on clinical presentation alone difficult [7].

Such findings reinforce the need for broad genetic screening approaches available within the public healthcare system to accurately diagnose patients within this diverse clinical spectrum [48, 49].

However, careful phenotypic assessment, targeted organ-specific evaluations, and consideration of differential diagnoses before genetic testing remain essential as they can (a) improve the quality and completeness of phenotypic data, (b) support the selection of the most appropriate genetic testing strategy, and (c) facilitate accurate interpretation of genetic variants once results become available [50, 51]. While thorough clinical assessment remains essential, our results support complementing phenotype-driven evaluation with genome-wide genetic testing, particularly when targeted testing is negative or when the phenotype is complex [52].

Finally, we acknowledge that revising a clinical diagnosis after genetic testing can be challenging for families [53]. When genetic testing later identifies an alternative molecular diagnosis associated with an overlapping phenotype, it can be difficult for families to understand the discrepancy between the initial clinical diagnosis and the subsequent genetic findings [53]. In some cases, this could lead to confusion or emotional stress, particularly if the newly identified condition is associated with a different prognosis, more severe outcomes, or additional late-onset manifestations [50, 53]. In such situations, careful genetic counseling plays a critical role in helping families understand the diagnostic process and the implications of the revised diagnosis [50, 53].

### Limitations

This study enhances the understanding of the complex genetic findings related to phen22q11.2. However, the limitations of the present study should be acknowledged.

In a considerable proportion of patients, no definitive molecular diagnosis could be established. Since not all patients with clin22q11.2 underwent identical or comprehensive genetic testing, some of this proportion is likely attributable to the study’s non-exhaustive nature.

Part of the study relied on retrospective data collection. However, not all patients underwent identical clinical assessments at presentation. Nor were all the missing data available in retrospect. These lacunae will necessarily compromise statistical comparisons of the patient categories considered.

Due to resource limitations, Fig. 1’s ”ES + CNV” analysis was performed in a subcohort of patients only. Selection of this subcohort (*n* = 88, chosen from *n* = 131) was not made on a random basis. Instead, inclusion was based on a stricter set of clinical criteria than had been applied in the initial phase of the study. This inconsistency further compromises the between-category comparisons mentioned in "Statistical analysis" section and "Phenotype comparison" section.

## Conclusions

The extensive genetic and phenotypic heterogeneity observed -together with a notable proportion of pathogenic variants located outside the 22q11.2 region compared to those within it- underscores the limitations of targeted genetic testing in clin22q11.2. In contrast, ES with CNV analysis offers a clear advantage by enabling the simultaneous detection of a wide range of genomic alterations that may be missed by more targeted testing methods, thereby improving diagnostic accuracy, reducing diagnostic delay, optimizing genetic counseling, and informing clinical management [48, 49]. Although CMA remains the recommended first-line diagnostic tool for suspected 22q11.2DS, growing evidence, including this study, supports the use of ES with CNV detection as a first-tier high-throughput diagnostic option, especially in patients with congenital anomalies [48, 49, 54].

## Supplementary Information

Supplementary Material 1.

## Data Availability

The datasets generated and/or analysed during the current study are available in the ClinVar and DECIPHER repository: https://www.ncbi.nlm.nih.gov/clinvar/, https://www.deciphergenomics.org/The datasets used and/or analysed during the current study are available from the corresponding author on reasonable request.

## Abbreviations

22q11.2DS: 22q11.2 deletion syndrome
clin22q11.2: Clinically suspected 22q11.2 deletion syndrome
CHD: Congenital heart disease
CMA: Chromosomal microarray analysis
CNV: Copy number variant
ES: Exome sequencing
FISH: Fluorescence in situ hybridization
GDD: Global developmental delay
ID: Intellectual disability
MLPA: Multiplex ligation-dependent probe amplification
phen22q11.2: Phenocopies of 22q11.2 deletion syndrome
SNV: Single nucleotide variant
VUS: Variant of unknown significance
