# Supplementary material for: Phenocopies of 22q11.2DS: revealing genetic diversity in clinically suspected 22q11.2 deletion syndrome
Source: Mol Cell Pediatr. 2026 Apr 30;13:24. doi: 10.1186/s40348-026-00236-1 (PMC13129039; doi:10.1186/s40348-026-00236-1)
Supplement: Supplementary file 1 — Supplementary Material 1. [file 40348_2026_236_MOESM1_ESM.docx]

**SUPPLEMENTARY MATERIALS**

| **Column A (Presence of ONE of the following)** | **Column B (TWO or more core features)** | **Column C (ONE core feature from B + ONE associated feature)** |
| --- | --- | --- |
| Conotruncal cardiac anomaly (e.g., tetralogy of Fallot, interrupted aortic arch, truncus arteriosus, major aorto-pulmonary collateral arteries) | Characteristic facial abnormalities | Long slender fingers and hands |
| Parent of an affected child | Non-conotruncal congenital cardiac defect | Short stature |
|  | Immunodeficiency or thymic hypoplasia | Hypotonia |
|  | Cleft palate, velopharyngeal insufficiency, or swallowing difficulty | Renal abnormalities or Potter sequence |
|  | Hypocalcaemia | Psychiatric (especially bipolar) disorders |
|  | Learning difficulties/developmental delay | Family history of congenital heart defects |

**Table 1. Tobias criteria for considering 22q11 deletion syndrome ^1^.**

Combinations of clinical features that should lead to consideration of FISH analysis for 22q11 deletion syndrome. FISH analysis should be performed in patients who meet one criterion in column A, two criteria in column B, or one criterion in column B and one criterion in column C.

| **Genetic Testing** | **Number of patients (n)** |
| --- | --- |
| Karyotype | 90 |
| Karyotype + ES + CNV analysis | 54 |
| Karyotype + FISH (other locus) | 30 |
| Karyotype + FISH (other locus) + ES + CNV analysis | 23 |
| Karyotype + FISH (other locus) + CMA | 8 |
| Karyotype + FISH (other locus) + other targeted NGS testing | 6 |
| Karyotype + CMA + ES + CNV analysis | 4 |
| Karyotype + FISH (other locus) + ES | 3 |
| Karyotype + other targeted NGS testing | 4 |
| Karyotype + CMA | 2 |
| Karyotype + FISH (other locus) + other targeted NGS testing +CMA + ES + CNV analysis | 2 |
| Karyotype + FISH (other locus) + other targeted NGS testing + ES + CNV analysis | 2 |
| Karyotype + ES | 2 |
| Karyotype + FISH (other locus) + CMA + ES + CNV analysis | 1 |
| Karyotype + FISH (other locus) + CMA + ES | 1 |
| Karyotype + other targeted NGS testing + CMA + ES + CNV analysis | 1 |
| Karyotype +other targeted NGS testing + ES + CNV analysis | 1 |
| Karyotype + other targeted NGS testing + ES | 1 |
| CMA | 2 |
| Other targeted NGS testing | 2 |
| CMA + ES | 1 |

**Table 2. Overview of genetic testing strategies performed in the study cohort following 22q11.2 targeted FISH and/or MLPA analysis in the light of phenotypic spectra.**

This table summarizes the genetic testing strategies applied in the cohort after the initial targeted evaluation for 22q11.2 deletion syndrome (22q11.2DS). All 336 patients with clinically suspected 22q11.2DS (clin22q11.2) first underwent targeted testing for 22q11.2 deletion. Following molecular confirmation of 22q11.2DS in 88 patients and their exclusion from further genetic analysis, 240 of the remaining 248 patients with clin22q11.2 underwent additional genetic investigations detailed in this table. Based on the diagnostic workflow described above, karyotyping was performed in 235 patients, FISH targeting loci other than 22q11.2 in 76 patients, other targeted NGS examinations in 19 patients, CMA in 22 patients, ES without CNV analysis in 8 and ES with CNV analysis in 88 patients.

CNV: Copy number variation; CMA: Chromosomal microarray analysis, ES: Exome sequencing, FISH: Fluorescence *in situ* hybridization; MLPA: Multiplex ligation-dependent probe amplification; NGS: next-generation sequencing

| **Patient ID** | **FISH probe** |
| --- | --- |
| FISH1 | DiGeorge II /10p14 deletion |
| FISH2 | BCR/ABL1/, ToTelVision Mixture 7 |
| FISH3 | Wolf-Horschhorn (WHSC1) |
| FISH4 | ABL1/BCR, Williams-Beuren |
| FISH5 | CHARGE (CHD7), ABL1/BCR |
| FISH6 | Wolf-Horschhorn (WHSC1) |
| FISH7 | ToTelVision Mixture 4 and 11, WCP 4, WCP 11 |
| FISH8 | ToTelVision Mixture 11 |
| FISH9 | Vysis LSI D13s319/13q34 FISH Probe, Williams-Beuren, DiGeorge II /10p14 deletion probe |
| FISH10 | Wolf-Horschhorn (WHSC1) |
| FISH11 | ToTelVision Mixture 1 |
| FISH12 | Williams-Beuren, DiGeorge II /10p14 deletion |
| FISH13 | 1q21/SRD(1p36), DiGeorge II /10p14 deletion |
| FISH14 | Vysis LSI D13s319/13q34 FISH Probe, TelVysion Mixture 6 |
| FISH15 | 1q21/SRD(1p36) |
| FISH16 | 1q21/SRD(1p36), DiGeorge II /10p14 deletion |
| FISH17 | SE 14/22, ToTelVision Mixture 9 |
| FISH18 | Williams-Beuren, SE 14/22, SE 13/21 |
| FISH19 | DiGeorge II /10p14 deletion , CEP8 |
| FISH20 | Williams-Beuren |
| FISH21 | SE 14/22 |
| FISH22 | CHARGE (CHD7), ABL1/BCR |
| FISH23 | DiGeorge II /10p14 deletion, CEP8, CHARGE (CHD7), GATA4, DSCR1 |
| FISH24 | CHARGE (CHD7) |
| FISH25 | GATA4,Wolf-Hirschhorn (WHSC1), ToTelVision Mixture 10 |
| FISH26 | GATA4 |
| FISH27 | Williams-Beuren |
| FISH28 | CHARGE (CHD7), subtel 18p, Cri du chat/CTNND2 |
| FISH29 | ToTelVison Mixture 12 |
| FISH30 | Prader-Willi/Angelman(SNRPN) |
| FISH31 | CHARGE (CHD7), SHOX |
| FISH32 | Wolf-Horschhorn (WHSC1) |
| FISH33 | CHARGE (CHD7) |
| FISH34 | SE X/SRY, ToTelvision Mixture 2 |
| FISH35 | Williams-Beuren |
| FISH36 | Williams-Beuren |
| FISH37 | ToTelvision Mixture 2, CHARGE (CHD7) |
| FISH38 | Williams-Beuren |
| FISH39 | Williams-Beuren |
| FISH40 | SE X |
| FISH41 | CHARGE (CHD7), SNRPN, DSCR1, 8p23, WCP18, P16, 9p + 9q, BCR/ABL1 |
| FISH42 | EVI1-3q26.2, ToTelvision Mixture 3, WCP 3 |
| FISH43 | Williams-Beuren |
| FISH44 | Vysis LSI D13s319/13q34 FISH Probe |
| FISH45 | CHARGE (CHD7) |
| FISH46 | WCP6, WCP15, ToTelVision Mixture 10 |
| FISH47 | GATA4 |
| FISH48 | DiGeorge II /10p14 deletion |
| FISH49 | ToTelVision Mixture 10 |
| FISH50 | CHARGE (CHD7) |
| FISH51 | ToTelvision Mixt12, ToTelvision Mixt15, WCP18 |
| FISH52 | DiGeorge II /10p14 deletion |
| FISH53 | Williams-Beuren |
| FISH54 | WCP14 |
| FISH55 | DiGeorge II /10p14 deletion |
| FISH56 | Williams-Beuren |
| FISH57 | Williams-Beuren |
| FISH58 | Williams-Beuren |
| FISH59 | Williams-Beuren |
| FISH60 | Williams-Beuren |
| FISH61 | Prader-Willi/Angelman(SNRPN) |
| FISH62 | CHARGE (CHD7) |
| FISH63 | Wolf-Horschhorn (WHSC1) |
| FISH64 | CHARGE (CHD7) |
| FISH65 | Wolf-Horschhorn (WHSC1), ATXN2L, ToTelvision Mix 4 |
| FISH66 | Williams-Beuren, PML/RARA |
| FISH67 | ToTelvision Mixture 4, WCP4 |
| FISH68 | Williams-Beuren, CDKN2A |
| FISH69 | DiGeorge II /10p14 deletion, ToTelVision Mixture11 |
| FISH70 | 14q11 – acro p arm |
| FISH71 | Wolf-Horschhorn (WHSC1), Prader-Willi/Angelman(SNRPN) |
| FISH72 | Prader-Willi/Angelman(SNRPN) |
| FISH73 | ToTelVision Mixture 14 |
| FISH74 | ToTelVision Mixture 7 |
| FISH75 | CEP8 |
| FISH76 | ToTelVision Mixture 7, Vysis LSI D13s319/13q34 FISH Probe |

**Table 3. FISH probes targeting genomic loci other than 22q11.2.**

| Gene | Variant | Protein | Forward primer (5’>3’) | Reverse primer (5’>3’) |
| --- | --- | --- | --- | --- |
| *NOTCH1* | c.1441G>A | p.GLY481Ser | CAGTGTCTGCAGGGCTACAC | AAGCAACCCACAGATGTTCC |
| *JAG1* | c.550C>T | p.Arg184Cys | TGGCAGACGCTGAAGCAGAACAC | AAGTTTTGTTGCCATTCTGGTCA |
| *TBX1* | c.438-2A>T |  | CTTCCACCAGCTAGGGTGAC | CCCTCACGCTTACAACCACT |
| *CHD7* | c.6157C>T | p.Arg2053Ter | TGTACAATGTAGAATGCCCTTGA | AGCTTGGCTGGCAGAGCTTA |
| *PHF8* | c.718C>T | p.Arg240Ter | AACCTTGTGGAGACACCGAAGA | CTGGCTGCCTCCAGAAGCCAT |
| *MYH7* | c.4145G>A | p.Arg1382Gln | AGCAGTACGAGGAGGAGACG | CAGAAGTCAGGCTGCTCAGA |
| *JAG1* | c.1080dup | p.Glu361Ter | GCCTGCCTCTCTGATCCCTGTC | CTCACCGAGACATTCACACTGG |
| *FLNC* | c.3070C>T | p.Gly1024Ter | GTGGGACAGGAACAAGCATT | GGGTGACCATCGTAGGTGAT |
| *ELN* | c.1055_1056del | p.Pro352ArgfsTer23 | TTAGTGCCTGGTGGGCCA | AGATGAGGGGATGAGACGTG |
| *KCNQ1* | c.1486_1487del | p.Leu496AlafsTer19 | TGTCCCCACACTTTCTCCTC | TGGGCACTAGGCGAGTAGAT |
| *FLNB* | c.2158G>A | p.Val720Met | CGCATTGACATCCAGATGAA | GGTGTATGACCAGGCATTTCCA |
| *CDK13* | c.382C>T | p.Gln128Ter | CTGGCGAGAGGCAAGAGG | CACATCCTCGTATTCCACCA |
| *CHD7* | c.3327G>C | p.Arg1109Ser | TCGAGTGATAAAGGGGTCCT | GCATCAAATTCTGAGCAACG |
| *FLNA* | c.2474A>T | p.Asp825Val | GTCAGCATCGGCATCAAGT | TGGTCAGCAAAGAGGACCAT |
| *CHD7* | c.3310_3311del | p.Ile1104Ter | TCGAGTGATAAAGGGGTCCT | GCATCAAATTCTGAGCAACG |
| *TBX1* | c.612C>G | p.Tyr204Ter | CTCCAGGTACGCCTTCCAC | CCGTTGTCGTCCAGTAGGTT |
| *SON* | c.5753_5756del | p. Val1918GlufsTer87 | AAGCGCAAAAGATCTCCAAAGC | GGGGAAATGCTAAAGCTCCTTC |
| *MED13L* | c.3401G>A | p.Cys1134Tyr | TCTCCGATTCCGTGATGAAT | AATCGCACTAAACCCACAGGT |
| *DSC2* | c.2398del | p.Ala800LeufsTer56 | GGCTTCACAACCCAAACTGT | TGTGCCACTCCGAGTAAGTG |
| *PDE4D* | c.1038T>G | p.Ile346Met | GGAAACATTTCCTGGAATATATAATGA | TCAGACTAGAGCTGTGCATCAA |
| *CHD7* | c.4505del | p.Ser1502Ter | CAGGTACAACAGCTTTCCAAGAA | CCCAATGCATCTTGTAAGCACTT |
| *TMEM260* | c.721dup | p.TYr241LeufsTer3 | TGTTCTTGAAGTTCCGAGATGA | ACCAGGCTGAAGGTTCCATA |
| *TMEM260* | c.942-10T>C |  | CCGAAATCTCTCTTTGCTCCTA | CCACACCCATGAAAAGTGGTT |
| *NOTCH1* | c.599G>T | p.Gly200Val | CAGGATGTCAACGAGTGTGGC | CAGCGGGCAGCACTACCT |
| *CNOT1* | c.439C>T | p.Gln147Ter | TTAACCCATTGTGGTGCAAAAG | TGCTATATCTTGGAAGCCACCT |
| *COL4A6* | c.1768G>A | p.Gly590Ser | CAAAGCATTGGTCACACTGGCT | CACGGGGTCCAGGAAGTCCT |
| *DSPP* | c.1531G>T | p.Glu511Ter | AACAGCAGTAGCCGAGGAGA | GTCATTGTCATCATTCCCATTGTTAC |
| *FBN1* | c.3209-2A>G |  | TGAAGAAAGGAACTGCACAGGT | CCCAAACTTACCCATGCAGTTC |
| *ZEB2* | c.656dup | p.Tyr220LeufsTer19 | CCCATTAGCTGCCATTGATT | TTCTTCTCGTGGCGGTACTT |
| *NR2F2* | c.103_109del | p.Gly35ArgfsTer75 | GACGCAGCCCCCATAGATA | TGTTGCTGCTGCTGCTGCTTG |

**Table 4.** **Primer sequence list used for Sanger sequencing.**

| primer | primer sequence | conc. (µmol/l) | product length (bp) |
| --- | --- | --- | --- |
| USP9X 18F | TTGATCGTCTGAAGGCTTCC | 0.2 | 179 |
| USP9X 18R | TCCTCATGATAATCACTGTCACA | 0.4 |  |
| USP9X 19F | GGAGTTTGTCTGCCTCAAGT | 0.2 | 199 |
| USP9X 19R | CGCCCACAAAGAGCTCAATT | 0.4 |  |
| **HSD17B3 11F** | CATTCCGCGGTAAACACCTC | 0.2 | 191 |
| **HSD17B3 11R** | CCTCCATCTTCAGCGGACTA | 0.4 |  |
| **USH2A 41F** | CCTTTCACCAGAGTCCCAGA | 0.2 | 278 |
| **USH2A 41R** | CCATGGGCTAAGAGCAGAAG | 0.4 |  |
| 6FAM-M13 | 6FAM-TGTAAAACGACGGCCAGT | 0.8 | - |

**Table 5. *USP9X*-QMPSF primer sequences (primers of control regions are in bold).**

1. Tobias ES, Morrison N, Whiteford ML, Tolmie JL. Towards earlier diagnosis of 22q11 deletions. *Arch Dis Child*. Dec 1999;81(6):513-4. doi:10.1136/adc.81.6.513
